# A Simple Method for Locating and Calibrating the Keyhole for the Retrosigmoid Approach using Ct Plain Scans

**DOI:** 10.2174/0115734056436600260605080616

**Published:** 2026-07-02

**Authors:** Jing Wu, Shenyu Li, Feng Wei, Guocheng Li, Jie Liu

**Affiliations:** 1 Department of Neurosurgery, The First People’s Hospital of Nanning, Nanning, Guangxi, China; 2 Department of Neurosurgery, The Second Affiliated Hospital of Guilin Medical University, Guilin, Guangxi, China; 3 Department of Neurosurgery, Guangxi Zhuang Autonomous Region Brain Hospital, Liuzhou, China; 4 Department of Neurosurgery, Hezhou People's Hospital, Hezhou, China

**Keywords:** Retrosigmoid approach, Frankfurt line, End point of the digastric groove, Theoretical keyhole, Calibrated keyhole, Junction of transverse sinus-sigmoid sinus

## Abstract

**Introduction/Background:**

The precise positioning of the keyhole is crucial for neurosurgical operations in the retrosigmoid approach. Although recent implementations like 3D Slicer localization, brain 3D-printed model localization, neuro-navigation, and augmented reality technology can perform individualized analysis and precise positioning, they come with high equipment requirements, complex procedures, and increased costs, significantly limiting their widespread use in primary healthcare institutions or primary hospitals in developing countries. It is necessary to introduce a method for using CT plain scans, combined with the relationship between the end point of the digastric groove and the Frankfurt line, to accurately localize and calibrate the keyhole for the retrosigmoid approach.

**Materials and Methods:**

Using data from CT plain scans combined with surface anatomical landmarks of the skull, based on the relationship between the end point of the digastric groove and the vertical line of the Frankfurt plane, which passes from it, the surgeon can determine preoperatively whether the keyhole position is reliable and optimize the final keyhole drilling position intraoperatively.

**Results:**

The 'theoretical keyhole' offers a safe and reliable landmark for locating the cranial projection of the posterior margin of the transverse-sigmoid sinus junction. This target is identified craniometrically *via* the vertical relationship between the posterior endpoint of the digastric groove and the Frankfurt horizontal plane. This directly determines the intraoperative drilling position. A few anatomic variations require optimized calibration, termed “calibrated keyhole”. A small subset of cases exhibit show “calibrated keyhole” being positioned rearward or forward compared to the “theoretical keyhole.”

**Discussion:**

Preoperative CT imaging data review and systematic evaluation of anatomical variations are prerequisites for individualized keyhole localization in the retrosigmoid approach, which is critical to optimizing surgical precision and minimizing procedural risks.

**Conclusion:**

Accurate intraoperative analysis of drilling location from CT data can improve safety and surgical efficiency in the retrosigmoid approach, reduce the risk of venous sinus injury, and reduce postoperative complications.

## 1 INTRODUCTION

The retrosigmoid approach is a classical neurosurgical technique for posterior cranial fossa surgery, widely employed to manage various lesions in the cerebellopontine angle [1]. Since this approach was proposed in 1905, numerous scholars have continuously refined and popularized it [2, 3]. A critical step in craniotomy *via* this approach is the accurate localization of the skull projection corresponding to the posterior edge of the transverse sinus-sigmoid sinus junction-referred to as the “keyhole”-which serves as the basis for craniotomy [4]. This precise localization facilitates adequate exposure of the cerebellopontine angle at the posterior edge of the sigmoid sinus while eliminating bony obstructions. During craniotomy, full utilization of the bony window space posterior to the sigmoid sinus and inferior to the transverse sinus is essential. If the bony window is too far from the target area, it may lead to excessive cerebellar retraction or even resection intraoperatively, resulting in limited exposure of the surgical field. Furthermore, overexposure of the transverse and sigmoid sinuses, despite failing to enhance effective surgical exposure, increases the risk of iatrogenic injury to these venous sinuses during bone removal.

Therefore, the optimal craniotomy strategy entails accurate localization of this critical keyhole, forming a bony window that precisely exposes the inferior edge of the transverse sinus and the posterior edge of the sigmoid sinus. Simultaneously, efforts should be made to preserve an intact bone flap to avoid excessive resection of the posterior cranial fossa bone [5]. Appropriate craniotomy techniques reduce the incidence of complications such as scalp hydrops, cerebrospinal fluid leakage, and intracranial infection secondary to bony defects.

Precise keyhole localization is pivotal for the success of retrosigmoid approach surgeries. Although numerous studies have explored this topic in depth, most rely on specific anatomical markers (*e.g*., osseous and muscular attachment sites), various measurement data, and empirical judgments. While these methods are generally applicable, they lack the capacity for individualized analysis. Recent advances, such as 3D Slicer-based localization, three-dimensional printed brain model-guided localization, neuronavigation, and augmented reality technology, enable individualized analysis and precise positioning. However, these techniques are associated with high equipment requirements, complex operational procedures, and increased costs, which significantly limit their widespread application in primary healthcare institutions or hospitals in developing countries [6-8].

Accordingly, this study explores the correlations between cranial Computed Tomography (CT) data, the “theoretical keyhole,” and the “calibrated keyhole,” aiming to achieve accurate, efficient, and straightforward keyhole localization for the retrosigmoid approach with low time consumption and minimal operational effort.

## 2 MATERIALS AND METHODS

All cranial CT imaging data from enrolled patients were rigorously protected, with all personal identifiers completely anonymized to comply with medical research confidentiality requirements. Intraoperative photographs, if involved in subsequent analysis, were also de-identified following the same protocol. The study was conducted in accordance with the Declaration of Helsinki and relevant national regulations. Written informed consent was obtained from all eligible participants; for incapacitated patients, consent was provided by their authorized legal surrogates. Ethical approval was granted by the Institutional Review Board (IRB) of Hezhou People’s Hospital (Approval No.: [HZPH-IRB-2025011979]).

This study aimed to investigate the universal relationship between skull anatomical localization, CT imaging characteristics, and the retrosigmoid approach in a broad population, rather than focusing on the clinical outcomes of a specific neurological disease. Therefore, no specific disease was restricted in the inclusion criteria to ensure the generalizability of the anatomical and imaging findings for the retrosigmoid approach, which could provide a universal reference for neurosurgical planning of this approach in clinical practice.

The inclusion criteria for this study were as follows: (1) patients aged 18-80 years with complete clinical data and high-quality CT imaging data sufficient for skull anatomical localization analysis; (2) patients who underwent cranial CT examination for neurosurgical evaluation (including retrosigmoid approach planning) in the Department of Neurosurgery between January 2024 and December 2024.

The exclusion criteria were as follows: (1) patients with skull fractures that may alter native skull anatomy; (2) patients with a history of craniocerebral surgery (within 5 years prior to enrollment) that could induce skull anatomical alterations; (3) patients with primary skull lesions (*e.g*., tumors, osteomyelitis) or craniofacial deformities interfering with anatomical localization; (4) patients with congenital intracranial vascular malformations that affect CT imaging interpretation for retrosigmoid approach planning.

To minimize selection bias, all patients were enrolled independently by two senior neurosurgeons who were blinded to each other’s decisions. Disagreements, if any, were resolved through consensus with a third senior neurosurgeon.

Before the localization of the keyhole, three definitions must first be clarified: Frankfurt line, theoretical keyhole, and calibrated keyhole.

(1) Frankfurt Line. The Frankfurt line was officially proposed at the Skull Measurement Conference in Frankfurt, Germany, in 1882 and finally confirmed in 1884. The Frankfurt line connects the infraorbital point, the superior border of the external auditory canal. In most cases, the external occipital protuberance is also on the same horizontal line. Typically, both points lie on a horizontal line, the baseline for dividing the cranium and the base of the skull, and serve as vital reference landmarks for the localization of neurosurgical procedures.

(2) Theoretical keyhole and calibrated keyhole. The keyhole in the retrosigmoid approach is the bone hole located at the skull projection of the posterior edge of the transverse sinus-sigmoid sinus junction. This hole is a symbolic key for craniotomy in the retrosigmoid approach [9, 10]. The end point of the digastric groove is the point of disappearance of the groove extending posteriorly and superiorly, *i.e*., the highest point before the disappearance of the digastric groove. The digastric groove is formed when the posterior belly of the digastric muscle travels medial to the mastoid process of the temporal bone and can be considered the path of contact between the muscle and the bone surface.

“Theoretical keyhole” is defined as the intersection between the Frankfurt line and a line vertical to it, passing from the end point of the digastric groove (Fig. **1a** and **b**). Theoretical keyhole is the projection on the skull surface for the posterior edge of the transverse sinus-sigmoid sinus junction [11, 12].

The theoretical keyhole is mostly located on the skull projection at the junction of the transverse sinus and sigmoid sinus, according to preoperative localization (Fig. **2**). There is no need to adjust the drilling position during surgery. However, in actual clinical surgeries, it’s found that there’s a certain distance from the skull projection at the junction of the transverse and sigmoid sinuses in a small proportion of cases. By analyzing the preoperative CT scans, the intraoperative keyhole of craniotomy in the retrosigmoid approach can be adjusted anteriorly or posteriorly to comfirm the final intraoperative drilling position, which is referred to as the “calibrated keyhole”.

Through analyses of images, anatomical studies, and surgical studies, we found that the calibrated keyhole largely overlaps with the theoretical keyhole. In a few cases, variation is to be expected. Although this variation rate is low, it dramatically affects the safety of surgery.

After clarifying these concepts, the relative positions of “theoretical keyhole” and “calibrated keyhole” can be measured on the preoperative image to guide drilling. The specific method can be divided into the following four steps.

### 2.1 Step 1: Find the Endpoint of the Digastric Groove on the CT Images

The digastric groove was identified on sequential transverse Computed Tomography (CT) images, and its posterior endpoint was localized by tracing the structure superiorly across adjacent axial planes (Figs. **3** and **4**).

### 2.2 Step 2: Location of the Theoretical Keyhole

After locating the endpoint of the digastric groove, maintain the mouse pointer stationary while scrolling the mouse wheel to view the upper plane where the transverse sinus turns into the sigmoid sinus in the transverse CT images. This rolling action is similar to making a line vertical to the Frankfurt line, which passes from the endpoint of the digastric groove.

The static digital cursor coordinate established within this plane represents the ideal geometric locus of the keyhole. This point can be indirectly localized using external cranial surface landmarks and is designated herein as the 'theoretical keyhole' (Fig. **5**).

### 2.3 Step 3: Clarify the Relative Position of the Theoretical Keyhole and the Actual Keyhole

To ensure optimal target accuracy, the alignment between the digital cursor and the cranial projection of the transverse-sigmoid sinus junction must be verified-a step critical for safe intraoperative navigation. When perfect spatial alignment is achieved, this coordinate-defined as the 'theoretical keyhole'-marks the precise trajectory for the surgical burr hole. Conversely, if a anatomical mismatch occurs, the linear displacement between the cursor and the sinus junction projection must be quantified preoperatively on the axial CT images to adjust the drill path. The real drilling hole, called “the calibrated keyhole,” can be calibrated from the theoretical keyhole preoperatively (Fig. **6**).

### 2.4 Step 4: Draw the Skull Landmarks and Locate the Drilling Keyhole During the Operation

After the patient was well positioned, draw the Frankfurt line, locate the endpoint of the digastric groove on the skin; then make a vertical line to the Frankfurt line, passing from the endpoint of the digastric groove. The intersection between two lines is the theoretical keyhole. Just drill a theoretical keyhole if it overlaps the CT-scan-identified skull projection of the transverse sinus-sigmoid sinus junction; if not, drill a calibrated keyhole.

Based on this, the required bone flap (dashed line) and arc-shaped skin flap are drawn as illustrated in Fig. (**7**).

Among the landmarks on the Frankfurt line, the infraorbital point and the upper edge of the external auditory meatus have constant anatomical positions that are easy to locate. On the other hand, some surgeons would like to use the external occipital protuberance to draw the Frankfurt line during surgery, combined with one of the two positioning points, because the the external occipital protuberance is just on the occipital bone and near the surgical zone. However, the external occipital protuberance often shows significant variations; some are large and prominent, while others are small and difficult to palpate. The position of the external occipital protuberance also exhibits clear anatomical variations, being higher in some cases and lower in others. Therefore, during surgery, it is impossible to directly draw the Frankfurt line based on the external occipital protuberance. Notably, the geometric principle that two points define a straight line is equally applicable to the analysis of cranial CT imaging data in this study-this principle underpins the precise construction of the Frankfurt line. Specifically, the Frankfurt line is defined as the line connecting the infraorbital point to the upper edge of the external auditory meatus and serves as a fundamental anatomical reference for skull base localization (Fig. **8**). This line is then extended backward to intersect with the midline, and the relative position relationship between this intersection point and the external occipital protuberance is assessed. If this intersection point coincides with the external occipital protuberance, the external occipital protuberance and the upper edge of the external auditory meatus can be used directly to form the Frankfurt line during surgery (Fig. **9**). In actual clinical practice, to achieve more accurate and efficient results, the author often uses the extension of the line connecting the infraorbital point and the upper edge of the external auditory meatus as the Frankfurt line, abandoning the reference to the external occipital protuberance, especially in surgical approaches where the external occipital protuberance has empirically been used as a reference marker, such as for the posterior longitudinal fissure and supracerebellar infratentorial approaches.

In the retrosigmoid surgery, to facilitate accurate intraoperative identification of the Frankfurt line, an ECG electrode sheet can be placed on the line, specifically anterior to the external auditory canal (Fig. **10**). Notably, tactile confirmation by palpating the ECG electrode sheet further enhances the precision of Frankfurt line localization. Additionally, the surgical drape should be positioned to reserve a small amount of extra space posteriorly (Fig. **10b**), ensuring the electrode sheet and the underlying Frankfurt line remain clearly visible and can be precisely localized even after the skin flap is incised.

## 3 LIMITATIONS AND TECHNICAL CONSTRAINTS OF CT-GUIDED KEYHOLE LOCALIZATION

### 3.1 Presence of Two or More Digastric Grooves

When evaluating transverse CT sections, anatomical duplications or duplications of the digastric groove may occasionally be observed (Fig. **11a** and **b**). To resolve this variation, independent theoretical keyholes must be modeled for each identified groove structure. The definitive surgical landmark is then selected by identifying the specific groove trajectory whose calculated keyhole exhibits the maximum spatial proximity to the posterior boundary of the transverse-sigmoid sinus junction, thereby establishing it as the primary intraoperative reference.

### 3.2 Offset of Scanning Baseline Inaccuracy

Notably, the selection of scanning baselines for cranial CT cross-sections also exerts a critical impact on keyhole localization accuracy. Commonly used baselines include the Eyebrow-Meatal Line (EML), orbitomeatal line (OML), and radiographic baseline (RBL) (Fig. **12a**). This study adopted the RBL as the preferred baseline for CT scanning, primarily because the RBL aligns most closely with the Frankfurt plane-anatomical consistency between the scanning baseline and the Frankfurt plane ensures that the reconstructed CT images accurately reflect the skull base anatomical structure, laying a solid foundation for precise localization of the theoretical keyhole (as shown in Fig. **12b**). In contrast, scans using the OML, particularly the EML, as a baseline resulted in significant deviations in both the measured distances of anatomical landmarks and the localization accuracy of the theoretical keyhole. These deviations stem from poor parallelism between the baselines and the Frankfurt plane, which distorts the spatial relationship of the transverse-sigmoid sinus junction on CT images. Therefore, effective communication with CT technicians prior to scanning is indispensable to confirm the adoption of the RBL and avoid baseline-related errors in subsequent keyhole calibration.

## 4 RESULTS

In clinical practice, the theoretical keyhole mostly overlaps the skull projection of the posterior edge of the transverse-sigmoid sinus junction. However, a few variations still pose a threat to patients’ safety [13, 14].

Preoperative detection of variations in venous sinus anatomy is critical to avoiding intraoperative complications. If the posterior margin of the transverse-sigmoid sinus junction lies well posterior to the predicted theoretical keyhole coordinate (Fig. **13a**-**d**), standard drilling risks direct sinus laceration during bone flap resection. In such cases, the definitive entry point must be modified with a posterior corrective shift, placing the actual burr hole behind the initial theoretical target.

When the skull projection for the posterior edge of the transverse sinus-sigmoid sinus junction is located in front of the theoretical keyhole as shown in Fig. (**14a**, **b**), if not recognized before surgery, it is difficult to find the posterior edge of the transverse-sigmoid angle promptly, requiring additional bone removal. This results in excessive bone defect during the return of the bone flap, not only prolonging the operation time but also increasing the likelihood of postoperative complications such as scalp hydrops, cerebrospinal fluid leakage, and even intracranial infection [15, 16]. At this point, it is necessary to calibrate and move forward. Based on the measured distance, make optimization adjustments. That means the calibrated keyhole is located in front of the theoretical keyhole.

Consequently, it is necessary to conduct careful, individualized image analysis to confirm whether the theoretical keyhole is safe and reliable, and whether calibration of the drilling position is necessary before the operation.

## 5 DISCUSSION

The retrosigmoid approach is a pivotal surgical route for treating cerebellopontine angle lesions [17]. The core of this craniotomy lies in accurately localizing the skull projection corresponding to the posterior edge of the transverse sinus-sigmoid sinus junction, defined as the “keyhole.” Precise drilling at this keyhole creates a bony window that exposes the posterior edge of the sigmoid sinus and the inferior edge of the transverse sinus, minimizing retrosigmoid bone flap defects and maximizing reduction of posterior cranial fossa bone obstruction to the surgical field [18, 19].

Historically, pioneering neurosurgeons, including Cushing, Dandy, House, Rhoton, and Yaşargil, executed foundational anatomical investigations that refined the retrosigmoid approach, ultimately establishing it as the definitive surgical corridor for managing cerebellopontine angle lesions [20, 21]. However, traditional keyhole localization mainly relies on anatomical landmarks such as the asterion, the mastoid process, and the superior nuchal line. Although these landmarks offer some reference value, they struggle to support individualized analysis when anatomical variations in the positions of the sigmoid and transverse sinuses are encountered, thereby posing potential surgical risks. With the development and application of emerging technologies such as three-dimensional reconstruction from MRI/CT, neuronavigation, and surgical robots, keyhole localization accuracy has been improved [22]. Nevertheless, these advanced techniques are accompanied by excessive complexity and high equipment requirements, hindering their widespread popularization [9].

This study analyzed plain CT scans and found that the precise drilling position of the keyhole in the retrosigmoid approach can be optimized by comparing the relative positions of theoretical keyholes and calibrated keyholes. This method not only features simple operation but also enables individualized analysis.

For large neurosurgical centers in developed countries, neuronavigation or AR-augmented visualization technology may provide greater guidance. However, many developing or underdeveloped regions lack such advanced equipment. In this context, the proposed method can efficiently utilize plain CT scan data through thorough preoperative review and analysis. By comparing the relative positions of the theoretical keyhole and the transverse sinus-sigmoid sinus junction, the actual intraoperative drilling position can be optimized (*i.e*., keyhole calibration).

Although anatomical variations at the transverse sinus-sigmoid sinus angle are relatively rare, they demand close attention during retrosigmoid craniotomy. Intraoperatively, posterior deviation of the calibrated keyhole is hazardous; failure to identify this variation preoperatively may easily lead to injury of the transverse or sigmoid sinus during bone flap milling, causing uncontrollable bleeding. Conversely, anterior deviation of the calibrated keyhole, if unrecognized preoperatively, can result in difficulty locating the transverse or sigmoid sinus, necessitating additional bone removal and excessive bony defects during bone flap repositioning. This not only significantly prolongs surgical time but also increases the risk of postoperative complications such as scalp hydrops and intracranial infection.

During retrosigmoid craniotomy, in addition to variations at the transverse sinus-sigmoid sinus angle, anatomical variants of the external occipital protuberance require careful monitoring. When the external occipital protuberance does not lie on the Frankfurt plane or is flattened, the Frankfurt plane should be constructed by connecting the infraorbital point to the upper edge of the external auditory canal and extending it posteriorly. This plane serves as the demarcation between the skull base and the craniofacial region, eliminating the need for localization based on the external occipital protuberance.

Notwithstanding the strengths of this study, it is not without limitations. First and foremost, the absence of follow-up surgical outcomes limits the comprehensive assessment of the proposed method’s clinical effectiveness; we were unable to correlate keyhole calibration with long-term postoperative outcomes such as complication rates and neurological function recovery. Secondly, this study is based on a single-center cohort, which may restrict the generalizability of our findings to diverse populations and anatomical phenotypes. Future studies should incorporate prospective multicenter designs with long-term follow-up to validate the efficacy and safety of this method and further explore its applicability in patients with complex sinus anatomical variations.

In conclusion, preoperative meticulous review of CT imaging, thorough analysis of potential anatomical variations, and individualized planning are indispensable for accurate keyhole localization in the retrosigmoid approach. The proposed calibration method based on plain CT data offers a practical alternative for institutions lacking advanced navigation technologies, with the potential to improve surgical safety and efficiency in resource-limited settings.

## CONCLUSION

In conclusion, the proposed methodology offers a streamlined, highly reproducible framework for keyhole localization in the retrosigmoid region. By optimizing baseline preoperative CT data alongside external craniometric landmarks, this technique accurately quantifies the spatial deviation between the theoretical and calibrated keyholes without relying on high-cost modalities like MRI-based neuronavigation, robotic stereotaxy, or complex 3D image fusion. The subsequent single-piece bone flap harvesting technique minimizes the incidence of dural venous sinus lacerations and related intraoperative complications. Given its minimal hardware footprint and rapid execution, this paradigm represents an efficient, cost-effective alternative with profound utility for community hospitals and resource-limited clinical settings globally.

## Figures and Tables

**Fig. (1):**
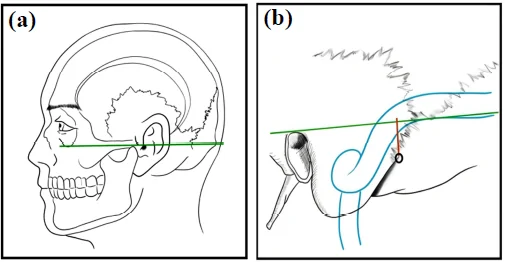
(**a**) Illustrates the Frankfurt line (green straight line), which connects the lowest point of the human orbit and the superior border of the external auditory canal, serving as the boundary between the skull base and the cranial brain (green straight line). (**b**) shows the intersection between the Frankfurt line and a line vertical to it, passing from the end point of the digastric groove (black line circle), which is defined as the “Theoretical keyhole” (black dashed circle), precisely located at the skull projection for the posterior edge of the transverse sinus-sigmoid sinus junction.

**Fig. (2):**
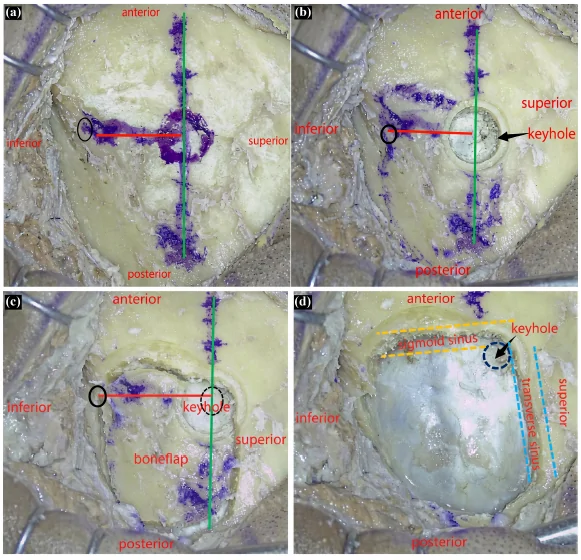
Illustrates the method for locating the keyhole of craniotomy in a retrosigmoid approach. (**a**) shows the intersection between the Frankfurt line and a line vertical to it, passing through the end point of the digastric groove (back line circle), referred to as the “theoretical keyhole.” (**b** and **c**) depict the craniotomy process using a retrosigmoid approach. (**d**) indicates that this “theoretical keyhole” precisely overlaps the skull projection of the posterior edge of the transverse sinus-sigmoid sinus junction, requiring no adjustment or optimization.

**Fig. (3):**
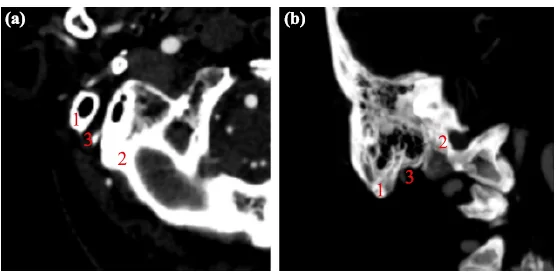
The localization of the digastric groove on CT. The digastric groove is the attachment of the posterior belly of the digastric muscle below the mastoid tip. This groove is wider at the beginning, with the mastoid tip on the lateral side and the squamous part above the occipital condyle on the medial side. (**a**) is a CT horizontal view demonstrating the digastric groove; (**b**) is a coronal view showing the digastric groove medial to the mastoid tip and lateral to the occipital scales. 1. mastoidale, 2. occipital bone, 3. digastric groove.

**Fig. (4):**
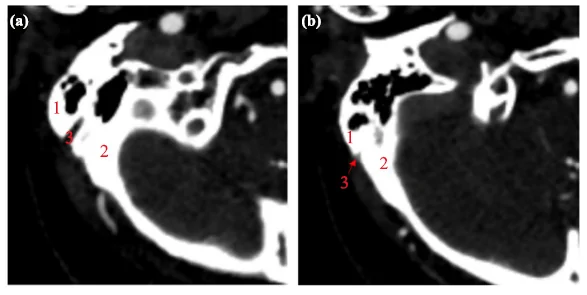
The localization of the endpoint of the digastric groove on CT. The digastric groove's endpoint is where it finally disappears; thin-layer CT continuous dynamic scanning can accurately locate it. (**a**) shows the digastric groove at a higher layer. (**b**) shows the upper layer before the disappearance of the digastric groove, which is the endpoint of the digastric groove (red arrow). 1. mastoidale, 2. occipital bone, 3. digastric groove.

**Fig. (5):**
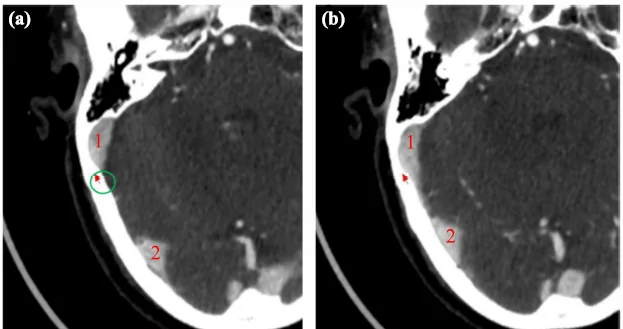
The layer of the posterior edge of the transverse sinus-sigmoid sinus junction on the CT. (**a**): The mouse pointer (*i.e*., the theoretical keyhole) is precisely at the posterior edge of the transverse sinus-sigmoid sinus junction shown on the CT (green circle), indicating that the theoretical keyhole (red arrow) is safe and reliable. (**b**) shows the transverse sinus on the next higher level. 1. sigmoid sinus, 2. transverse sinus.

**Fig. (6):**
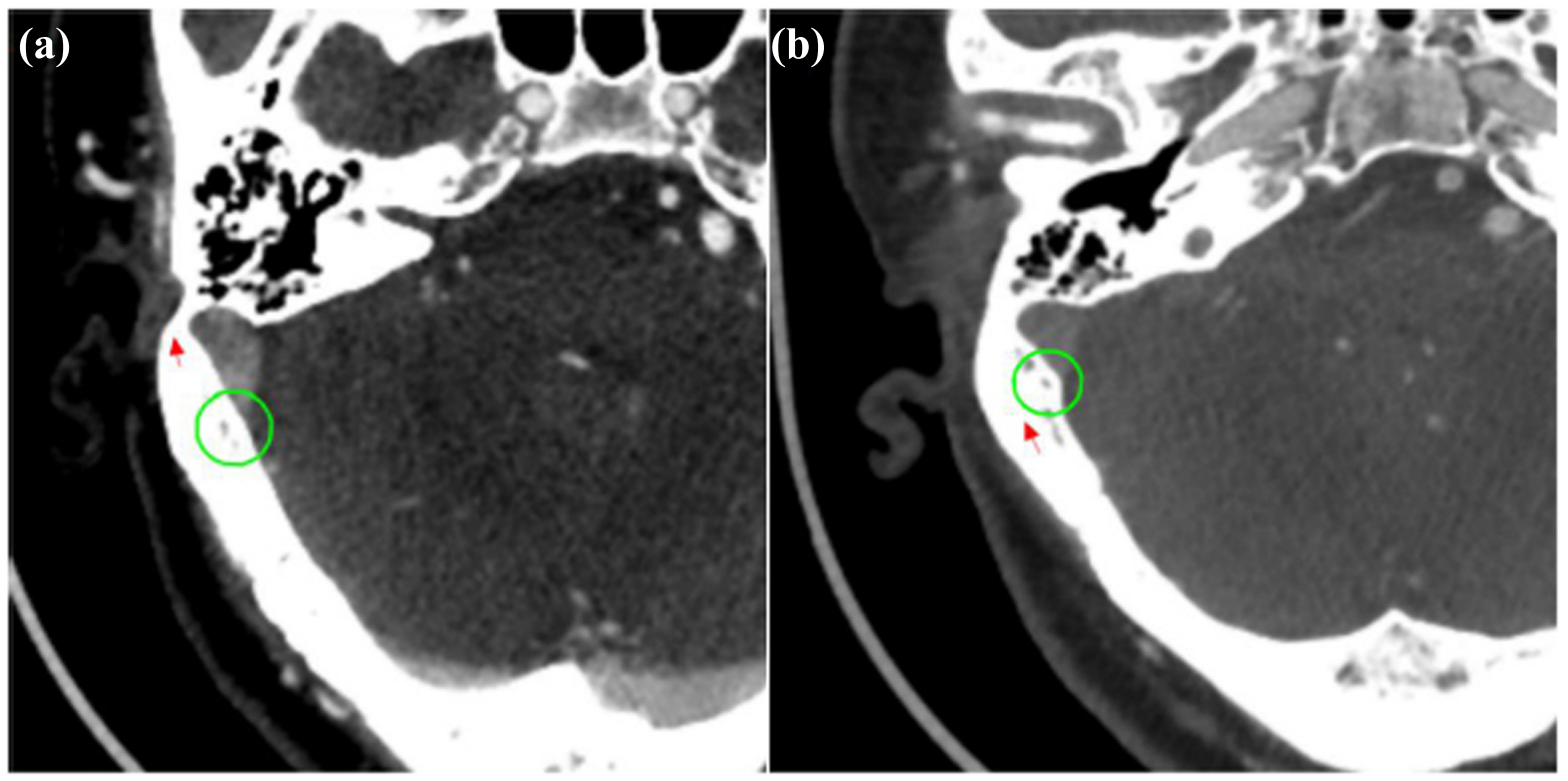
Preoperative modification of the drilling trajectory to account for anatomical variants of the transverse-sigmoid sinus junction. The structural target (green circle) is shown significantly displaced relative to the initial theoretical keyhole coordinate (red arrow). (**a**) Modification required for a posteriorly displaced sinus junction; the actual surgical burr hole site (green circle) is adjusted posteriorly relative to the baseline reference coordinate (red arrow). (**b**) Modification required for an anteriorly displaced sinus junction; the actual burr hole site (green circle) is shifted anteriorly.

**Fig. (7):**
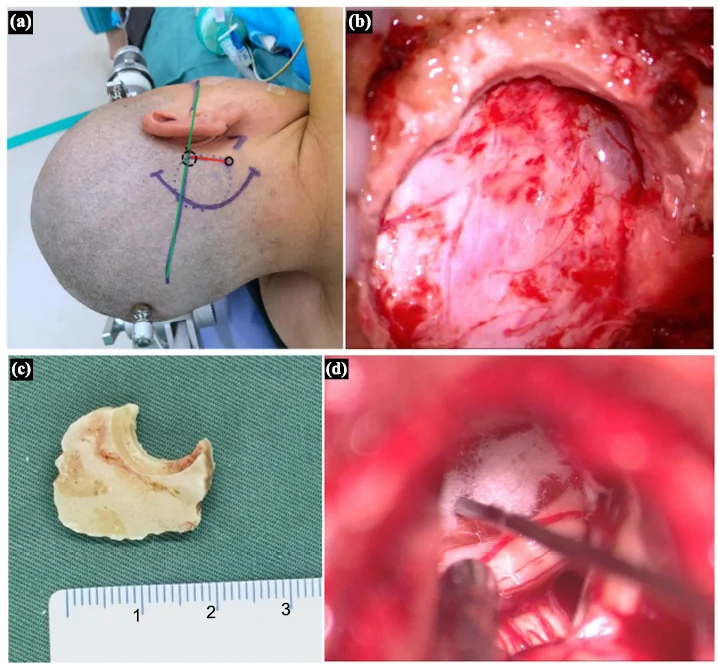
Surgical workflow for a retrosigmoid craniotomy guided by the theoretical keyhole. (**a**) Surface landmarking illustrating the Frankfurt line (green line) alongside the localization of the digastric groove endpoint and the theoretical keyhole coordinate, verified *via* preoperative CT to safely overlay the venous sinus junction. (**b**) Post-craniotomy view showing clean exposure of the transverse-sigmoid sinus margins based on the targeted keyhole trajectory. (**c**) Morphological evaluation displaying the dimensions of the harvested suboccipital bone flap. (**d**) Intraoperative visualization of the subsequent trigeminal nerve decompression.

**Fig. (8):**
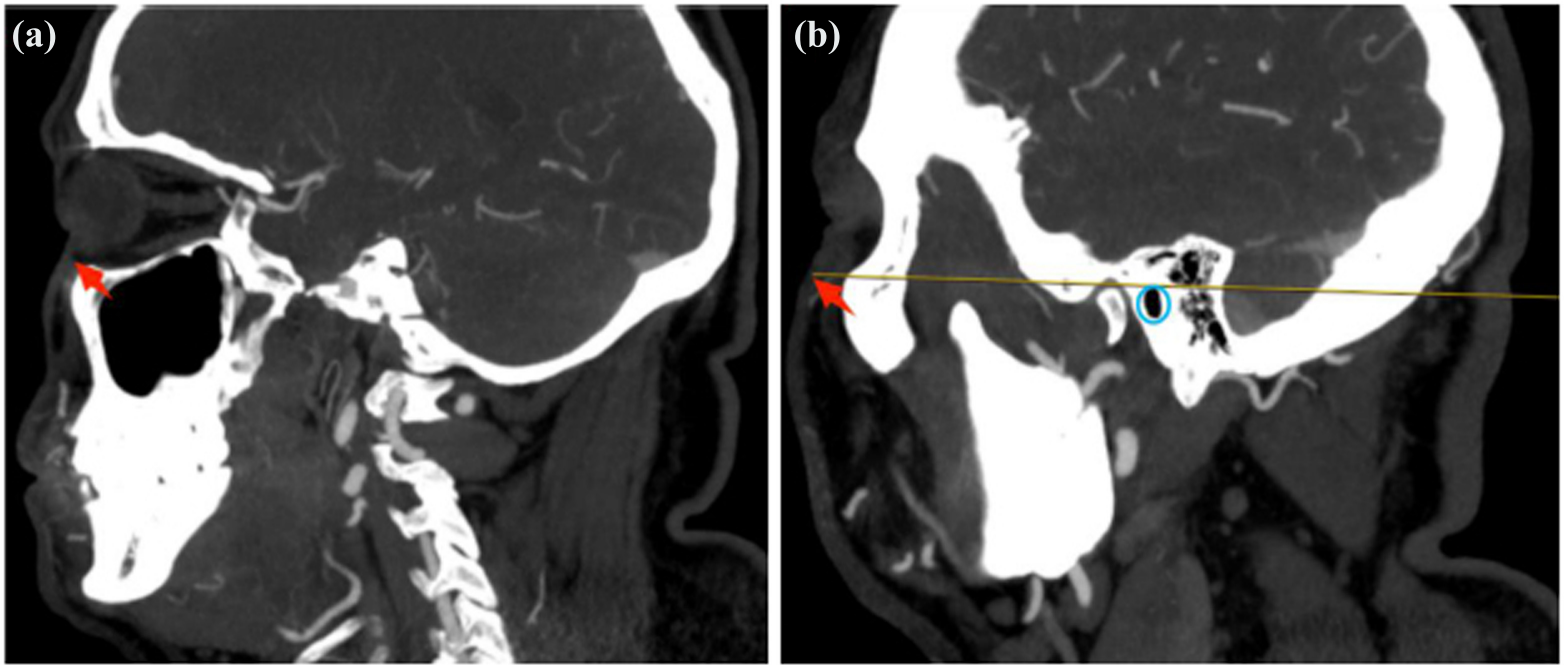
Multiplanar CT mapping of the Frankfurt line relative to the external occipital protuberance. (**a**) Identification of the lowest point of the margo infraorbitalis (red arrow) on a sagittal CT section. (**b**) Delineation of the Frankfurt horizontal plane. By utilizing a static axial reference point at the inferior orbital margin and evaluating the lateral sagittal planes, a line is projected connecting the margo infraorbitalis to the superior edge of the external auditory canal (blue circle).

**Fig. (9):**
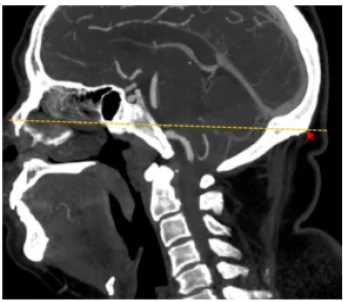
Midsagittal CT view confirming the position of the external occipital protuberance. By tracking the reference coordinates relative to the horizontal plane (yellow line) onto the median sagittal section, the relationship of the occipital landmarks is assessed. The external occipital protuberance (red arrow) aligns perfectly with the path of the Frankfurt line.

**Fig. (10):**
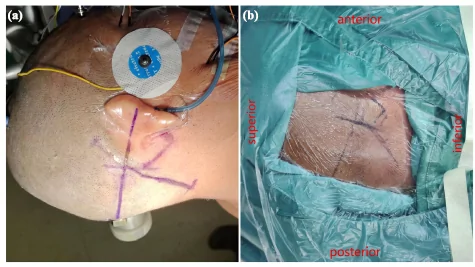
Method for maintaining landmark visibility during patient positioning and draping. (**a**) An adhesive electrode patch is fixed to the skin along the Frankfurt horizontal line, anterior to the external auditory meatus, to secure an anatomical marker before draping. (**b**) The sterile surgical drapes are positioned to leave a clearance margin posteriorly. This modification ensures the Frankfurt line remains identifiable and accessible for spatial orientation after the surgical incision is completed.

**Fig. (11a, b):**
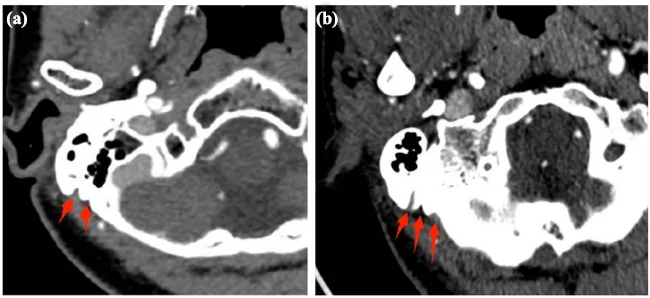
Digastric groove variation.

**Fig. (12):**
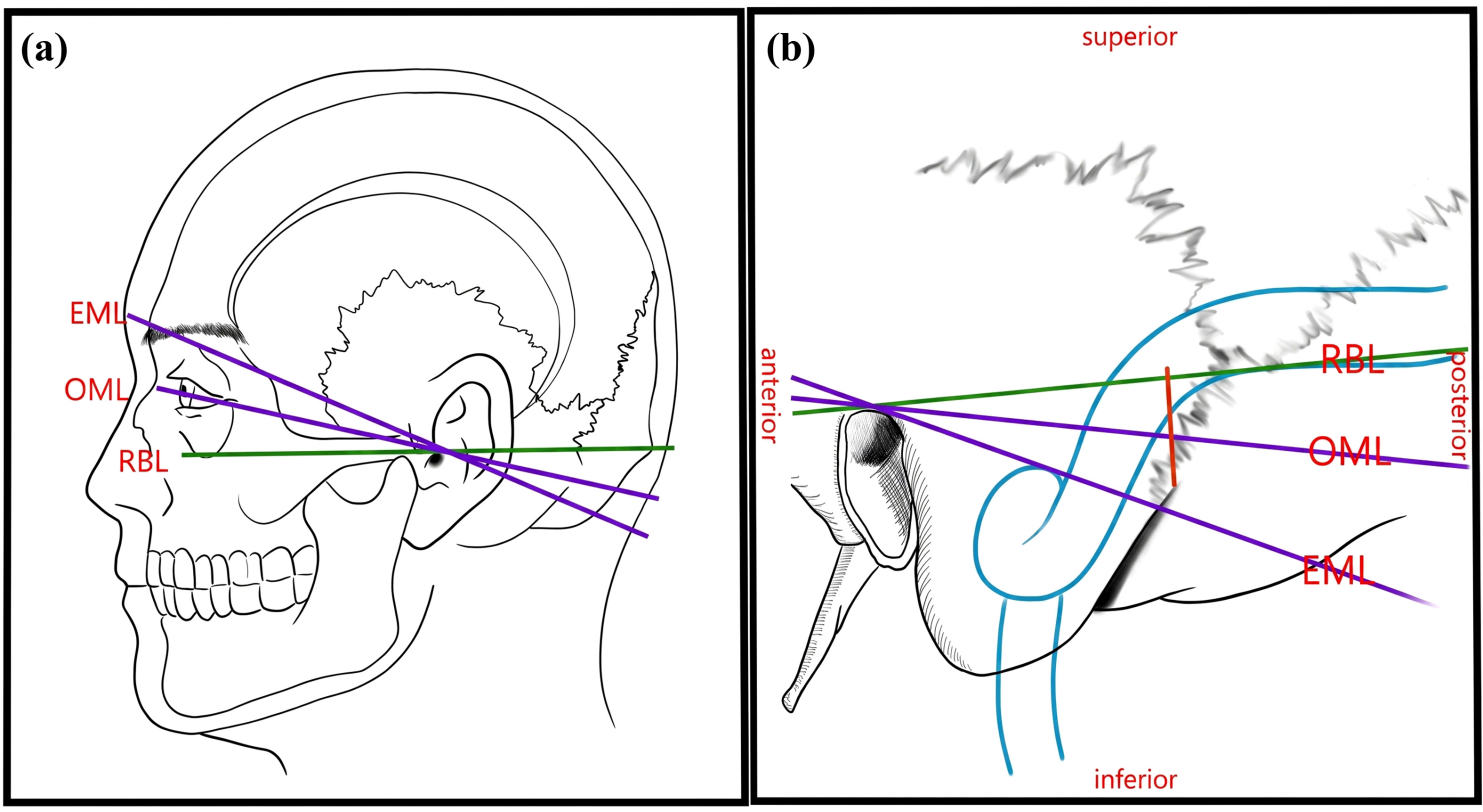
Influence of computed tomography (CT) acquisition planes on the geometric accuracy of keyhole localization. (**a**, **b**) Comparative schematic of standard cranial tomographic planes: the infraorbitomeatal line (IOML, historically RBL), the orbitomeatal line (OML), and the glabellomeatal line (GML, historically EML). The localization protocol defined herein utilizes the IOML as the primary scanning baseline. Utilizing planes with greater cranial angulation, such as the OML or GML, introduces significant translational displacement and systematic errors during the spatial projection of the theoretical keyhole.

**Fig. (13):**
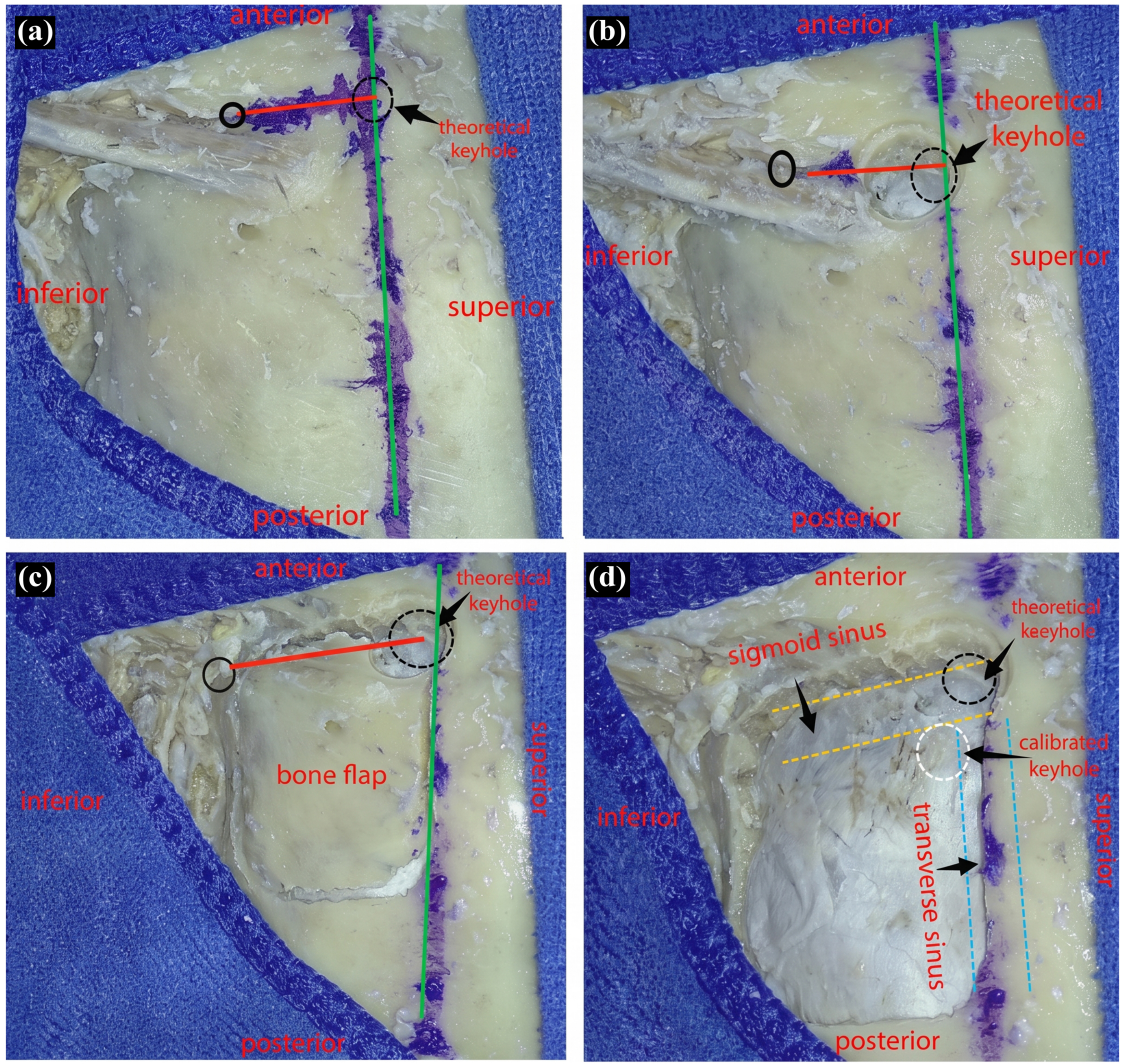
Schematic simulation demonstrating the structural conflict associated with uncorrected theoretical keyhole drilling in a posteriorly displaced sinus variant. (**a**) Projection of the initial theoretical keyhole coordinate (black dashed circle), determined by dropping a perpendicular vector from the Frankfurt plane (green line) to intersect the terminal endpoint of the digastric groove (black solid circle). (**b**, **c**) Simulation of a standard craniotomy executed blindly over the unadjusted theoretical keyhole trajectory. (**d**) Intraoperative mapping reveals the actual margin of the transverse-sigmoid sinus junction. Due to localized posterior displacement of the venous sinus, the safe surgical target-defined as the calibrated keyhole (white dashed circle)-lies posterior to the initial reference coordinate, demonstrating the high risk of sinus laceration if an entry point adjustment is omitted.

**Fig. (14):**
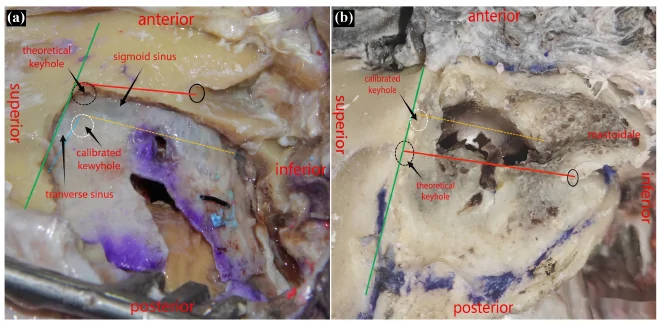
Cadaveric specimens demonstrating spatial divergence between the baseline theoretical keyhole and the calibrated surgical targets. (**a**) Specimen exhibiting a pronounced posterior deviation of the safe drilling zone; the calibrated keyhole coordinate is situated well posterior to the initial theoretical landmark. (**b**) Specimen demonstrating an anterior deviation; the calibrated target lies anterior to the baseline reference point. For both structural variants, failure to preoperatively map these atypical sigmoid sinus configurations *via* CT reconstructions significantly escalates the risk of inadvertent venous laceration and subsequent intraoperative complications.

## Data Availability

The data and supporting information are available in the article.

## LIST OF ABBREVIATIONS

CT: Computerized Tomography
3D: Three-dimensional
MRI: Magnetic Resonance Imaging
AR: Augmented Reality
PACS: Picture Archiving and Communication System
EML: Eyebrow-Meatal Line
OML: Orbitomeatal line
RBL: Radiographic baseline
